# Role of Late Gadolinium Enhancement in the Assessment of Myocardial Viability

**DOI:** 10.7759/cureus.22844

**Published:** 2022-03-04

**Authors:** Dr. Viraj Shah, Dr. Tushar Kalekar, Dr. Arunima Gupta, Dr. Purnachandra Lamghare

**Affiliations:** 1 Radiology, Dr. D Y Patil Medical College, Hospital and Research Centre, Pune, IND

**Keywords:** myocardial infarction, myocardial viability, late gadolinium enhancement, coronary revascularization, cardiac mri

## Abstract

Background: Prior to any revascularization procedure for coronary artery disease, it is essential to identify viable myocardium which will likely benefit from it. In such a situation, delayed enhanced cardiac MRI is beneficial.

Methods: Our study consisted of 50 patients with at least a one-month prior history of myocardial infarction (MI), abnormal findings on electrocardiography (ECG), and 2D-echocardiography (2D-ECHO), who were subjected to cardiac MRI performed on a 3T MRI machine. The MRI scans were evaluated for anatomical and especially functional characteristics of the heart, such as wall motion. On late gadolinium enhancement (LGE), the diseased segments were classified into two categories: < 50% LGE (viable) and > 50% LGE (non-viable).

Results: Of the 378 diseased segments detected on LGE, 137 (36.2%) segments showed < 50% LGE and 241 (63.8%) segments showed > 50% LGE. The segments showing < 50% LGE showed normokinesia or hypokinesia, with none of the segments showing akinesia or dyskinesia, whereas the segments showing > 50% LGE showed akinesia or dyskinesia predominantly. This was found to be statistically highly significant (p-value < 0.001).

Conclusion: Delayed enhanced-cardiac magnetic resonance (DE-CMR) imaging in patients with ischemic heart disease (IHD) helps evaluate the severity of the infarcted myocardium by classifying the diseased myocardium into viable and non-viable, as viable myocardium is more likely to regain functional recovery than non-viable myocardium. It also predicts the functional recovery of the myocardium after revascularization therapy.

## Introduction

Cardiovascular disease (CVD) remains the primary cause of mortality worldwide [1]. Indians are affected by CVD at least a decade before the rest of the world [2]. With the introduction of revascularization techniques such as percutaneous coronary intervention (PCI) and coronary artery bypass grafting (CABG), observations have been made on their effect on the reversibility of left ventricular (LV) dysfunction [3].

However, revascularization will not be helpful for all patients. Echocardiography establishes the hypokinetic or akinetic myocardium as diseased. This diseased myocardium may be stunned and hibernating or fibrosed. The viable myocardium is described as hibernating or stunned. Due to decreased perfusion, it is hypokinetic or akinetic [4].

After adequate revascularization, this hibernating and stunned myocardium is likely to regain normal myocardial activity, whereas the infarcted myocardium is less likely to recover [5-7]. In addition, pre-operative assessment of the infarct size can predict the functional recovery of dysfunctional myocardial segments after coronary revascularization [8-10].

Various techniques are used to assess myocardial viability in patients with ischemic heart disease (IHD). These techniques include echocardiography, 201Tl single-photon emission computed tomography (SPECT), fluoro-2-deoxyglucose (FDG) positron emission tomography (PET), and cardiac MRI.

Cardiac MRI is a non-invasive, non-ionizing technique that is used for the evaluation of progression of infarcted segments, thinning of LV wall, LV volume, LV shape distortion, and compensatory hypertrophy of healthy myocardium [11]. Cardiac MRI also helps to assess the level of perfusion in the tissue (micro-vascular status) [12]. Cardiac MRI offers more spatial and temporal resolution than other modalities. Cine MRI provides dynamic imaging of cardiac wall motion similar to echocardiography but with better endocardial border definition and superior wall motion assessment. Also, cardiac MRI does not involve ionizing radiation, unlike many other modalities like CT [13]. Unlike PET and SPECT, cardiac MRI provides quantitative cardiac viability, function, and perfusion information.

Infarct evaluation using contrast-enhanced CMR is a relatively novel approach. Extracellular contrast material reveals substantial signal strength in the infarcted areas on T1 weighted images. It is most likely that the extracellular contrast agent disperses into the intracellular space due to the breakdown of the myocyte membrane. Chronic infarcts have collagenous scars with increased interstitium between collagen fibers, leading to a higher contrast concentration and hence contrast enhancement [14]. Due to the revolutionary inversion recovery gradient echo sequence, infarcted myocardium may now be observed with much better resolution than spin-echo sequences [15].

In this study, we have highlighted the importance of LGE in delineating viable from non-viable myocardium, which will affect the selection of patients for revascularization procedures and predict the recovery of LV function. 

## Materials and methods

Patient selection

This was an observational study comprising 50 patients conducted in the radiology department. Before starting the study, an Institutional Ethical Committee (IEC) clearance was obtained at Dr. DY Patil Medical College, Hospital and Research Center, Pune, with approval number IESC/PGS/2019/168.

Patients ranging in age from 20 to 80 years were referred from the department of cardiology with at least a one-month prior history of myocardial infarction (MI), ECG findings of either ST-segment elevation myocardial infarction (STEMI) or non-ST segment elevation myocardial infarction (NSTEMI), abnormal 2D-echocardiography (2D-ECHO) findings, and were medically treated with thrombolysis. Patients who had undergone revascularization therapies like CABG surgery were excluded from the study. A thorough medical history was collected to rule out any potential contraindications to MRI, like metallic objects, pacemakers, and cochlear implants. Written informed consent was obtained from the patients before the MRI. Before the commencement of the MRI scan, each patient was given a brief explanation of the procedure. 

Imaging technique

A Magnetom Vida 3T MRI (Siemens, Erlangen, Germany) was used for the MRI evaluation. MRI was performed on patients supine (head first) with optimal placement and body stabilization. A standard Body Array 18 channel coil was used to acquire images. The participants were given light music to reduce the amount of noise in the MRI room. In addition, patients were trained for breath-holding (taking a breath, breathing out, and then not holding breath for 10-15 s). 

First, localizer sequences are done in various planes to localize the heart for anatomy and position. Pre-contrast scanning is done for morphology and function. The sequences used for morphology are T2 TRUFI, T2 HASTE Dark Blood, T2 TSE Dark Blood, T1 TSE Dark Blood, T2 STIR Dark Blood while 2 Chamber CINE TRUFI in Long Axis, 4 Chamber CINE TRUFI in Long Axis, CINE TRUFI Retro in Short Axis, CINE LVOT, and CINE RVOT are used to assess cardiac function. Contrast studies are done for perfusion and viability studies with dimeglumine gadopentetate (0.1 mmol/kg body weight). For perfusion, we used the DYNAMIC TRUFI SR EPAT sequence in 50 phases in 2-chamber, 4-chamber, and short-axis for the left side and a 2-chamber view for the right side of the heart. Following viability imaging, TI SCOUT is taken in the short axis after approximately 10 min of contrast injection to assess TI value. This TI value is further used for the delayed TRUFI high-resolution PSIR sequence. Except for CINE imaging, all image acquisitions were electrocardiogram (ECG)-gated (retrospective gating). 

The MRI images were analyzed for the structural anatomy of the heart as well as its functional aspect. 

Imaging evaluation

**Figure 1:**
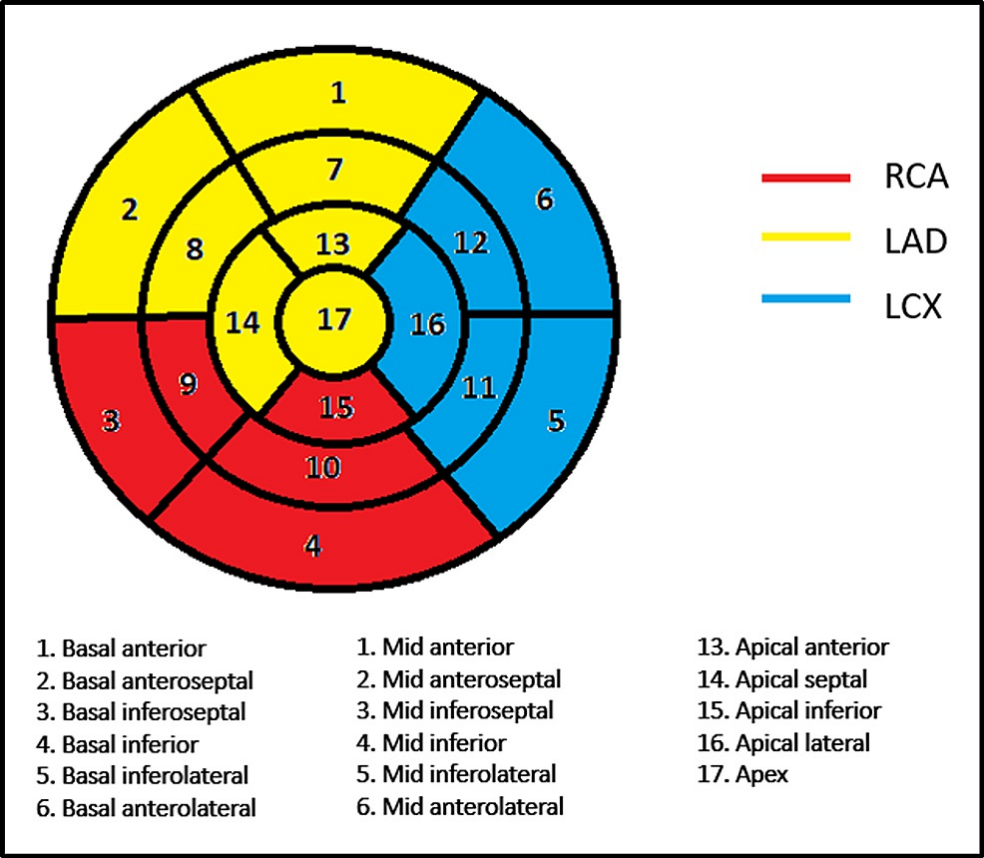
Seventeen segment LV model with the vascular territory. LV, left ventricle

The American Heart Association's 17-segment LV model [16] was utilized to identify segments with late gadolinium enhancement (LGE) (Figure 1).

**Figure 2:**
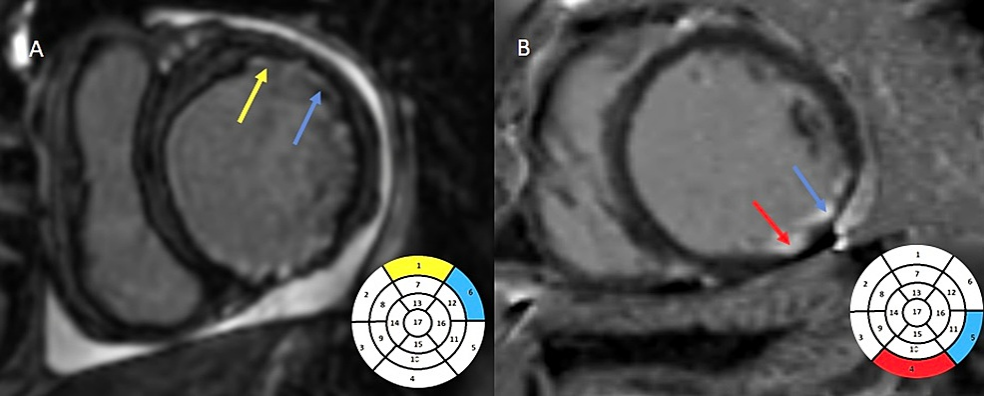
Delayed post-contrast-enhanced imaging on short axis demonstrating subendocardial (<50%) LGE. A: Subendocardial (<50%) LGE involving the anterior (yellow arrow - LAD territory) and antero-lateral segments (blue arrow - LCX territory). B: Subendocardial (<50%) LGE involving the inferior (red arrow - RCA territory), and inferolateral segments (blue arrow - LCX territory). LGE, late gadolinium enhancement; LAD, left anterior descending artery; RCA, right coronary artery; LCX,  left circumflex artery

**Figure 3:**
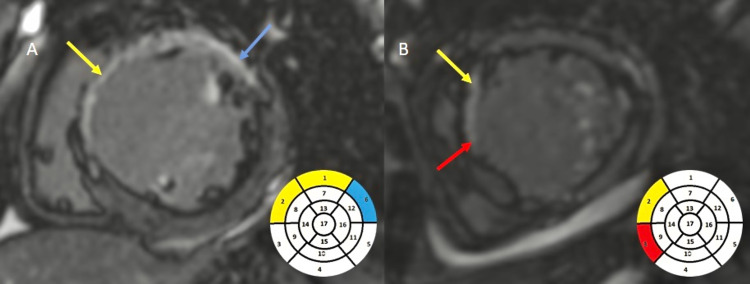
Delayed post-contrast-enhanced imaging on short axis demonstrating transmural (>50%) LGE. A: Transmural (>50%) LGE involving the anterolateral (blue arrow - LCX territory), anterior, and anteroseptal segments (yellow arrow - LAD territory). B: Transmural (>50%) LGE involving the anteroseptal (yellow arrow - LAD territory) and inferoseptal segments (red arrow - RCA territory). LGE, late gadolinium enhancement; LAD, left anterior descending artery; RCA, right coronary artery; LCX,  left circumflex artery

Based on the extent of LGE, we divided the diseased myocardium into segments showing <50% LGE (Figure 2) and >50% LGE (Figure 3) of the myocardial thickness based on visual eyeballing. A total number of diseased segments, the number of segments with less than 50% and the number of segments with more than 50 % enhancement, wall motion abnormalities, and distribution into the three vascular territories were presented as frequencies and proportions. The degree of wall motion abnormality was assessed based on visual "eyeballing" into one of the three categories in increasing order of severity (hypokinesia, akinesia, and dyskinesia). Normal wall motion was labeled as normokinetic. The distribution of segments was done into three vascular territories: left anterior descending (LAD), LCX (left circumflex), and RCA (right coronary) arteries.

 

Statistical analysis

Data were entered into Microsoft Excel, and statistical analysis was carried out in SPSS software version 17.0 (IBM, Armonk, NY). Qualitative variables like gender, LV clot, and microvascular obstruction (MVO) were presented as frequency and percentages. In addition, age and ejection fraction were categorized and represented as percentages. Bar diagrams and pie charts were used for the graphical representation of data. 

The Mann-Whitney U test and Kruskal-Wallis test were used to compare the number of segments diseased between gender and age categories. A Chi-square test was used to compare wall motion abnormality and the degree of LGE.

A p-value of <0.05 was considered statistically significant. 

## Results

Some 50 patients with 850 myocardial segments and 150 coronary territories were evaluated. Thirty-four (68%) were males; the mean age of the cases was 54.04 years which ranged from 30 to 77 years. The mean ejection fraction was 30.11% (14.7), with a lowest of 12% and a highest of 67%.

On DE-CMR, out of the total 850 segments, 378 segments showed DE-CMR (diseased myocardial segments), while the remaining 472 segments did not show any enhancement (normal myocardial segments).

The number of diseased segments was found to be higher in males than females, which was found to be statistically significant (p-value = 0.02). A comparison of the number of diseased segments in different age groups was statistically not significant (p-value of 0.202).

The diseased segments were divided into those that showed <50% LGE and those that showed >50% LGE (Table 1). Among the segments showing <50% LGE, 52 (38%) segments showed involvement of LAD territory, 43 (31.4%) segments showed involvement of LCX territory, whereas 42 (30.7%) segments showed involvement of RCA territory. Among the segments showing >50% LGE, 178 (73.9%) segments showed involvement of LAD territory, 27 (11.2%) segments showed involvement of LCX territory, whereas 36 (14.9%) segments showed involvement of RCA territory. 

**Table 1:** Distribution of diseased segments. LGE, late gadolinium enhancement

Diseased segments	Number	Percentage
Less than 50% LGE	137	36.2
More than 50% LGE	241	63.8
Total	378	100.0

Based on visual eyeballing, out of the 137 segments showing <50% LGE, 121 (88.3%) segments showed hypokinesia, 16 (11.7%) segments showed normal wall motion, and none of the segments showed akinesia or dyskinesia. Out of the 241 segments showing >50% LGE, 14 (5.8%) segments showed hypokinesia, 162 (67.2%) segments showed akinesia, and 65 segments (27%) showed dyskinesia. None of the segments showed normal wall motion. Thus, the segments showing <50% LGE showed normokinesia or hypokinesia, with none showing akinesia or dyskinesia, whereas the segments showing >50% LGE showed akinesia or dyskinesia predominantly. This was found to be statistically highly significant (p-value<0.001). Hence, as the degree of LGE increases, the severity of wall motion abnormality also increases. A segment with <50% LGE is considered a viable segment, whereas a segment with >50% LGE is considered non-viable (Table 2). 

**Table 2:** Comparison of wall motion abnormality and percentage of LGE. LGE, late gadolinium enhancement

Wall motion abnormality	Less than 50% LGE		More than 50% LGE	
	n	%	n	%
Normokinesia	16	11.7	0	0
Hypokinesia	121	88.3	14	5.8
Akinesia	0	0	162	67.2
Dyskinesia	0	0	65	27.0
Total	137	100.0	241	100.0
Chi-square p value=<0.001 (highly significant)				

**Figure 4:**
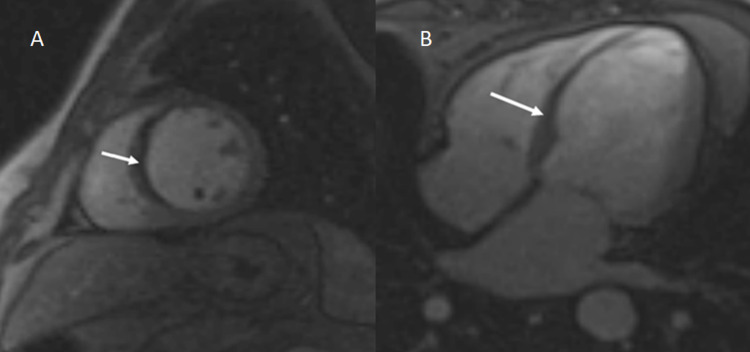
A - Short axis view; B - Four chamber view demonstrating a hypointense signal (white arrow) on first-pass perfusion involving the interventricular septum, i.e., MVO. MVO, microvascular obstruction

**Figure 5:**
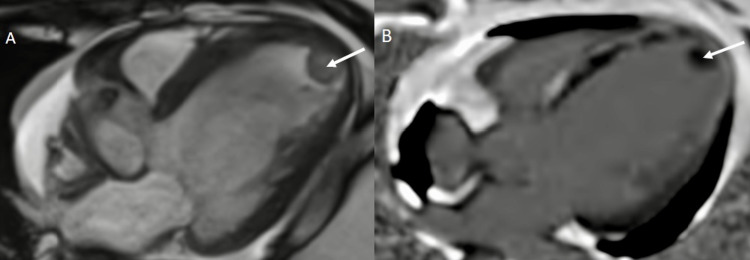
A: Four-chamber CINE TRUFI demonstrating a hypointense filling defect (white arrow) in the apical cavity of the LV. B: Delayed post-contrast-enhanced imaging in four-chamber view in the same patient demonstrating the non-enhancing hypointense filling defect (white arrow) within the LV as an LV clot. LV, left ventricle

Some 10 (20%) cases showed the presence of MVO in the LV wall (Figure 4). Some 18 (36%) of the study subjects were found to have LV clots (Figure 5). 

## Discussion

Myocardial viability before any revascularization technique is an essential clinical issue in individuals with IHD. Among the different modalities used in assessing myocardial viability, delayed enhanced-cardiac magnetic resonance imaging (DE-MRI) is a well-known MRI method for assessing viability [17]. The purpose of DE-MRI is to generate pictures with a strong contrast between diseased cardiac tissue that accumulates excess gadolinium (after IV treatment) and normal tissue that has a low gadolinium level [18]. The degree of transmural enhancement can predict the outcome of revascularization, which cannot be done effectively by other methods like 2D-ECHO, PET, and SPECT [19-20].

In our study, which involved 50 patients, 36.2% of the diseased segments showed <50% LGE and 63.8% showed >50% LGE, whereas in a similar study by Aggarwal et al. [21], consisting of 40 patients; 80% of the diseased segments showed less than 50% LGE, and 20% of segments showed more than 50% LGE. 

**Figure 6:**
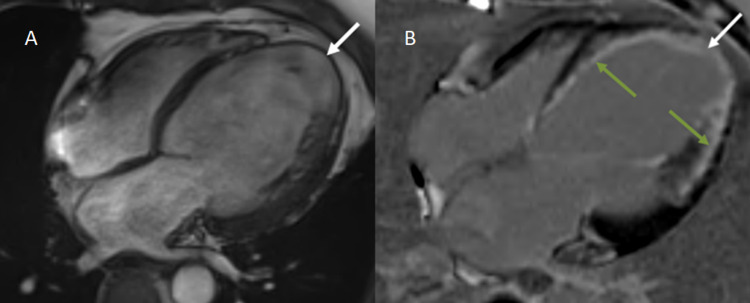
A: Four-chamber CINE TRUFI sequence depicting mild cardiomegaly with dilated left atrium and LV. Severe thinning of the cardiac apex (1 mm) with ballooning is also noted (white arrow). B: Delayed post-contrast enhanced imaging in the four-chamber view in the same patient demonstrates transmural (>50%) LGE in the apex (white arrow) and subendocardial (<50%) LGE involving the interventricular septum and lateral wall of the LV (green arrows). LGE, late gadolinium enhancement; LV, left ventricle

The two most important indicators of ventricular function recovery following revascularization operations are the degree of infarct as estimated by LGE and the end-diastolic wall thickness (EDWT) (Figure 6) [21]. According to research by Schinkel et al., segments having LV EDWT < 6 mm rarely exhibited contractile reserve, whereas most of the segments with comparably maintained EDWT of ≥ 6 mm showed contractile reserve [22]. Amado et al. found a strong link between histology and the delayed enhancement approach in an animal model (r2 = 0.94, p = 0.001) [23]. In 2008, Krittayaphong et al. examined the predictive efficacy of LGE and EDWT as measured by MRI in predicting restoration of LV function following CABG. The study revealed that LGE and EDWT are distinct predictors of functional recovery following revascularization. LGE appears to be of greater significance than EDWT [24]. 

Additionally, some investigations have hypothesized that delayed enhancement can effectively illustrate the amount of infarct, outcome, and occurrence of potential complications following revascularization. In 2016, Lee et al. assessed the effect of delayed gadolinium enhancement on long-term outcomes in patients undergoing CABG and found that the amount of LGE is a significant predictor of unfavorable cardiovascular events, regardless of LV function [25]. Lim et al. reported in 2018 that the amount of myocardial viability as measured by cardiac magnetic resonance LGE appeared to distinguish individuals who benefit differently after CABG [26]. Choi et al. evaluated 24 patients, seven days following satisfactory revascularization. The extent of the transmural infarct was negatively related to the myocardial contractile recovery [27]. Kim et al. conducted the first study of CE-CMR in 50 patients with chronic ischemia. The amount of diseased but viable myocardium proper to revascularization was related to more extensive improvements in wall motion and ejection fraction post-revascularization [28].

There were 10 (20%) study subjects in which microvascular obstruction (MVO) was detected in our study. In addition, these patients had severe LV systolic dysfunction. In the setting of acute MI, MVO may occur, which is a "no-reflow" process, indicating an inability to reperfuse a section of myocardium despite re-establishment of patency of epicardial coronary arteries. MVO is seen on MRI as a dark, non-enhancing region of the myocardium in both early and delayed enhancement [20]. Microvascular blockage implies significant ischemia (typically transmural) and is linked to a worse prognosis, adverse remodeling [29].

## Conclusions

Cardiac MRI is one of the most accurate tools in a radiologist's arsenal for assessing ventricular mass and volumes, aberrations of the wall motion, and ventricular function, both systolic and diastolic. With the use of DE-CMR in patients with IHD, it is possible to know the severity and extent of the infarcted myocardium, classifying the diseased myocardium into viable and non-viable, which helps with patient selection for revascularization therapy as viable myocardium is more likely to regain functional recovery after revascularization than non-viable myocardium.
